# Comparison of diagnostic performance of [^68^Ga]-Ga-FAPI-46 and [^18^F]-FDG PET/CT imaging for the detection of lesions and disease staging in patients with breast cancer

**DOI:** 10.22038/aojnmb.2024.80845.1573

**Published:** 2025

**Authors:** Kiana Radmehr, Saeed Farzanefar, Mehrshad Abbasi, Yalda Salehi, Najme Karamzade-Ziarati, Alireza Emami-Ardekani, Reyhaneh Manafi-Farid, Nasim Vahidfar, Davood Beiki

**Affiliations:** 1 Department of Nuclear Medicine, Imam Khomeini Hospital Complex, Tehran University of Medical Sciences, Tehran, Iran; 2 Research Center for Nuclear Medicine, Shraiati Hospital, Tehran University of Medical Sciences, Tehran, Iran; † These two authors contributed equally to this study.

**Keywords:** [^68^Ga]-Ga-FAPI-46, [^18^F]-FDG, Breast cancer, PET/CT

## Abstract

**Objective(s):**

To compare the diagnostic performance of [^68^Ga]-Ga-FAPI-46 and [^18^F]-FDG PET/CT imaging for the detection of lesions and disease staging in breast cancer.

**Methods:**

Twelve female patients with breast cancer (mean age= 49.2±13.29 years) and previous [^18^F]-FDG PET/CT were recruited in the study. [^68^Ga]Ga-FAPI-46 imaging performed in all patients within one month after [^18^F]-FDG PET/CT imaging. The acquired PET/CT data with both tracers were reconstructed. Tracer avid lesions with each PET tracer were identified and the semi-quantitative parameters i.e. SUV_max_, lesion counts and target-to-background ratio (TBR_max_) were analyzed.

**Results:**

Physiologic distribution of [^68^Ga]-Ga-FAPI-46 was observed in the liver, blood pool and kidneys, whereas no tracer uptake was noted in the brain and heart. The mean liver SUV_max_ for [^68^Ga] Ga-FAPI-46 was 1.5±0.1 which was lower than that noted for [^18^F]-FDG PET/CT (2.9±0.2). Likewise, the mean blood pool SUV_max_ value for [^68^Ga]-Ga-FAPI-46 was lower than [^18^F]-FDG PET/CT (1.7±0.1 versus 2.0±0.1). [^68^Ga]-Ga-FAPI-46 PET/CT demonstrated higher tracer uptake in the lesions detected in the brain, bone, internal mammary and lymph nodes in 4/12 patients. The overall lesions detections and the mean SUV_max_ values did not differ significantly between the two techniques. On the other hand, [^68^Ga]-Ga-FAPI-46 demonstrated higher mean TBR_max_ than [^18^F] FDG PET/CT particularly for lesions detected in kidneys, chest wall, mediastinum, and musculoskeletal lesions. However, both techniques offered identical TNM staging.

**Conclusion:**

The findings of this preliminary study demonstrated that [^68^Ga]-Ga-FAPI-46 and [^18^F]-FDG PET/CT offered identical disease staging in the breast cancer patients. [^68^Ga]-Ga-FAPI-46 showed lower liver and blood pool uptake and an enhanced tumor-to-background ratio, thereby suggesting its potential for improved lesions detection. This may open opportunity for emerging FAP based radioligand for therapeutic applications in advanced stage breast cancers. However, this needs validation in a larger number of patients.

## Introduction

 Breast cancer is the most common cancer among women worldwide, with an estimated 2.3 million new cases diagnosed in 2020 (1). Early detection and accurate diagnosis of breast cancer are crucial for effective treatment and improved patient outcomes. Imaging techniques, such as positron emission tomography/computed tomography (PET/CT), play an indispensable role in the diagnosis and staging of breast cancer by providing both functional and anatomical insights into the tumor, aiding accurate diagnosis and treatment planning.

 Fluorodeoxyglucose ([^18^F]-FDG) PET/CT, the most prevalent imaging modality for diagnosing and staging breast cancer, reports sensitivity and specificity ranging from 48–96% and 73–100%, respectively (2-5). [^18^F]-FDG, a glucose analog, accumulates in areas of high metabolic activity, particularly within cancer cells. However its uptake is not specific only to cancer cells but also observed in inflammatory and infectious sites, leading to potential false positives (6). Moreover, specific breast cancer types, such as invasive lobular or ductal carcinoma in situ and low-grade/low proliferation tumors, might exhibit negligible [^18^F]-FDG uptake, resulting in false negative results (6).

 In addition to increasing glucose consumption by tumor cells, tumor stroma comprises extracellular matrix proteins and specialized connective tissue cells, notably the activated cancer-associated fibroblasts (CAFs). Several studies advocate that these stromal cells significantly influence tumor initiation, progression, and metastasis. CAFs dominate the tumor stroma landscape, especially in breast cancer cases (7, 8).

 Fibroblast activation protein (FAP), a type II membrane-linked glycoprotein from the dipeptidyl peptidase IV protein family, expresses predominantly in CAFs across various epithelial carcinomas. This protein, present in many malignant tumors, aids in tumor cell migration, invasion, and angiogenesis (9, 10). Therefore, [^68^Ga]-Ga-FAPI, a novel radiotracer targeting FAP, was engineered to visualize tumor stroma. It has proven efficacious in detecting numerous tumors and metastases due to its proper targeting, rapid renal clearance, and high tumor-to-background ratio (7-10). 

 The novel PET/CT tracer, [^68^Ga]-Ga-FAPI, designed for cancer imaging, targets fibroblast activation proteins (FAP) that are overexpressed in various cancer types', inclusive of breast cancer (7-9). [^68^Ga]-Ga-FAPI PET/CT has shown promising preliminary results in the diagnosis and staging of various cancers, including breast cancer (10). Several studies have reported better diagnostic yield of [^68^Ga]-Ga-FAPI compared to [^18^F]-FDG PET/CT in breast cancer lesions (11-14). 

 Distinct from [^18^F]-FDG, [^68^Ga]-Ga-FAPI is highly specific to cancer cells and has shown a higher sensitivity for the detection of breast cancer lesions (11, 13). However, the clinical utility of [^68^Ga]-Ga-FAPI PET/CT in the diagnosis and staging of breast cancer is still under investigation. Further studies are needed to determine the optimal use of [^68^Ga]-Ga-FAPI PET/CT in breast cancer patients and to compare its diagnostic performance with other imaging modalities. In this preliminary study, we compared the diagnostic potential of [^68^Ga]-Ga-FAPI-46 and [^18^F]-FDG PET/CT for detection of lesions and disease staging in breast cancer patients.

## Methods

***Study design and participants***

 The study was approved by the ethics committee of Tehran University of Medical Sciences (IR.TUMS.MEDICINE.REC.1400.348). A written and informed consent was obtained from all patients following a detailed oral and written explanation. The study included 12 patients with confirmed breast cancer, who were referred for [^18^F]-FDG PET/CT either for metastatic work up, treatment response assessment or re-staging.

 Patients who did not give an informed consent, patients with unstable medical conditions (e.g. life-threatening arrhythmias), other malignancy or active inflammatory/ infectious disease, psychiatric illness and pregnant or lactating women were excluded from the study. Furthermore, patients with any intervention (surgery, biopsy or treatment) after FDG-PET/CT and before FAPI-46 imaging were excluded from the study.

 [^68^Ga]Ga-FAPI-46 PET/CT imaging was performed within one month after [^18^F]-FDG PET/CT in all patients.

 The day before scan, pathology results and conventional imaging findings such as MRI and CT were reviewed to confirm the diagnosis of breast cancer and assess any abnormalities. 

***Patient preparation***

 Patients were kept 6 hours fasting for performing [^18^F]-FDG PET/CT ensuring that blood glucose levels remained below 200 mg/dL. No specific preparation was required for [^68^Ga]-Ga-FAPI PET/CT imaging.

***PET/CT acquisition***

 [^68^Ga]-Ga-FAPI-46 and [^18^F]-FDG were injected intravenously where the injected activity varied from 3.3-8.4 mCi and 6.0-10 mCi respectively. [^68^Ga]-Ga-FAPI-46 was supplied by Pars Isotope (Tehran, Iran) in volume ranging from 10.0-15.0 cc and activity from 5.0-10.0 mCi per vial. PET/CT acquisition was performed about 60-min (50.0-70.0 min) after tracer injection using time of flight (TOF), GE Discovery IQ PET/CT scanner (GE, Health care, WI, USA). The whole body PET/CT scanning was performed with 3-min acquisition per bed position. Low -dose CT acquisition (40-120 mAs, 120 kev, matrix- 512×512, slice thickness 3.7 mm, rotation- 0.5 sec) was performed for attenuation correction and anatomical localization of the lesions. Attenuation, dead time, random events and scatter correction were applied to the reconstructed imaging data. 

 The reconstruction was done by using OSEM algorithm with 4-iterations/12 sub-iterations along with resolution recovery, Gaussian filter recovery methods (FWHM=6.4 mm). The reconstructed images were saved in 192×192 format for interpretation and analysis.

***Image interpretation***

**Figure 1:**
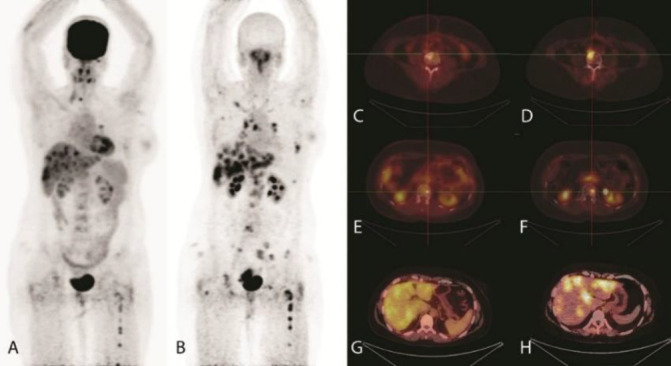
A 35-year-old female, known case of right sided breast cancer. [^18^F]FDG and [^68^Ga]Ga-FAPI-46 PET/CT MIP images (**A** and **B**, respectively) demonstrate numerous liver and bone metastases. [^68^Ga]Ga-FAPI-46 images demonstrate more radiotracer avid bone lesions (**D**, **F**), which were not present or were less visible in the [^18^F]FDG scan (**C**, **E**). Also, Lesion-to-liver ratio was higher in [^68^Ga]Ga-FAPI-46 (**H**) scan as compared with [^18^F]FDG (**G**)

**Figure 2:**
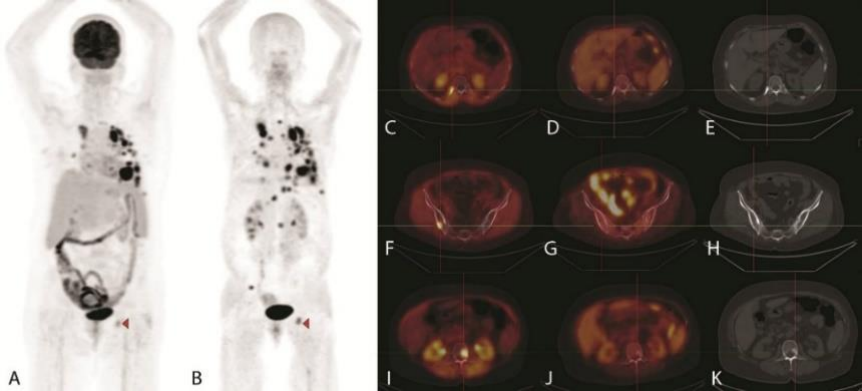
A 59-year-old female, known case of breast cancer, status post lumpectomy and chemoradiotherapy. [^18^F]FDG and [^68^Ga]Ga-FAPI-46 MIP images (**A** and **B**, respectively) demonstrate multiple lung and mediastinal lymph node metastases. The noteworthy point was the presence of only one hypermetabolic bone lesion in the left ischium in the [^18^F]FDG images (**A**, **arrow head**), while in the [^68^Ga]Ga-FAPI-46 images, more extensive bone metastases are seen, including left ischium (**B**,** arrow head**), right 12^th^ rib (**C**, with corresponding [^18^F]FDG PET/CT fusion and CT images, **D** and **E**), right iliac bone (**F**, with corresponding [^18^F]FDG PET/CT fusion and CT images, **G** and **H**), L2 vertebra (**I**, with corresponding [^18^F]FDG PET/CT fusion and CT images, **J** and **K**)

 Any uptake higher than background was considered abnormal. The analysis included the number, location, size, SUV_max_ and target -to-background ratio of the identified tracer avid lesions in each patient. A comparative analysis of [^18^F]-FDG PET/CT and [^68^Ga]-Ga-FAPI PET/CT data was performed. Representative [^18^F]-FDG and [^68^Ga]-Ga-FAPI PET/CT scans in two patients are presented in Figures 1 and 2 respectively.

***Statistical analysis***

 Data collection and analyses were done using IBM SPSS Statistics for Windows, Version 26.0. Armonk, NY: IBM Corp. Quantitative metrics were presented as mean and standard deviation, and qualitative data reported in the form of frequency and percentage. Comparative findings were analyzed using the Paired T-test and correlation coefficient. A p-value of ≤ 0.05 was the threshold for statistical significance.

## Results

***General characteristics***

 The mean age of the patients was 49.4±13.3 years. Nine (9/12) patients have undergone prior breast surgery. All patient have received chemotherapy. The patients were referred for [^18^F]-FDG PET/CT imaging for metastatic workup, treatment response evaluation or disease re-staging. The histopathological analysis documented estrogen receptor (ER) positivity in 7 (58.3%), progesterone receptor (PR) positivity in 5 (41.7%), and HER-2 positivity in 2 (16.7%) patients. The average Ki-67 index was 35.0±23.7%. The detailed results are presented in Table1.

**Table 1:** Characteristics of studied patients

**Variable**		**Mean±SD / n (%)**
Age		49.4±13.3
Treatments	Surgery	9 (75.0)
	Chemotherapy	12 (100.0)
Indications	M staging	7 (58.3)
	Restaging	2 (16.6)
	Response to therapy	3 (25.0)
Receptor status	ER*	7 (58.3)
	PR*	5 (41.7)
	HER2*	2 (16.7)
Ki-67 (%)		35±23.7

*ER: Estrogen Receptor, HER2: Human Epidermal Growth Factor Receptor 2, PR: Progesterone Receptor

***Background blood pool and liver uptake***

 The mean blood pool SUV_max_ for [^68^Ga]-Ga-FAPI PET/CT was 1.7±0.1 which was significantly lower than 2.0±0.1 observed for [^18^F]-FDG PET/CT (p=0.016). Likewise, the mean liver SUV_max_ value was also significantly lower in [^68^Ga]-Ga -FAPI PET/CT than [^18^F]-FDG PET/CT (1.5±0.1 versus 2.9±0.2 respectively, p=0.0001).

***Number for detected lesions***

 The mean number of detected lesions was 34.1±12.9 in [^68^Ga]-Ga-FAPI-4 and 32.2±12.8 in [^18^F]-FDG PET-CT scan, representing comparable results (p=0.074). There was no significant difference in the number of patients with detected lesions in different regions of the body between the two scans (Table 2).

**Table 2:** Number of patients with detected lesions in different body regions in [^68^Ga]-Ga-FAPI-46 versus [^18^F]-FDG PET/CT scans

**Body region**	**[** ^68^ **Ga]-Ga-FAPI PET/CT**		**[** ^18^ **F]-FDG PET/CT**		**p-value**
	**Number of patients with detected lesions**	**Median(range) of detected lesions/ body region**	**Number of patients with detected lesions**	**Median(range) of detected lesions/ body region**	
Brain	1	0 (0-3)	0	-	0.339
Head and neck	4	0 (0-7)	5	0 (0-7)	0.339
Lung	6	1 (0-29)	6	1 (0-29)	0.339
Mediastinum	8	4 (0-10)	8	2.5 (0-10)	1.00
Chest wall	6	0 (0-6)	5	0 (0-6)	0.339
Axillary	5	0 (0-6)	5	0 (0-6)	1.00
Liver and biliary system	3	0 (0-50)	3	0 (0-50)	1.00
Spleen	1	0 (0-12)	1	0 (0-12)	1.00
Pancreas	1	0 (0-1)	0	0 (0-1)	0.339
Adrenal	2	0 (0-2)	2	0 (0-2)	1.00
Gastrointestinal tract	4	0 (0-10)	4	0 (0-10)	1.00
Genitourinary tract	1	0 (0-1)	1	0 (0-1)	1.00
Pelvis	1	0 (0-12)	2	0 (0-12)	0.339
Musculoskeletal system	8	9 (0-52)	8	7 (0-52)	0.339

***Uptake intensity in lesions***

 The mean SUV_max_ value of the lesions in [^68^Ga]-Ga-FAPI-46 and [^18^F]-FDG PET-CT scans were 9.9±5.6 and 10.9±8.4, respectively (p=0.565). No significant difference was observed in the SUV_max_ values in different regions of the body between the two scans (Table 3).

**Table 3:** Average SUV_max _(Mean±SD) of the detected lesions in different body regions in [^68^Ga]-FAPI-46 versus [^18^F]-FDG PET/CT scans

**Body region**	**Average SUV**_max_ ** (Mean±SD)**		**p-value**
	**[** ^68^ **Ga]-Ga-FAPI-46**	**[** ^18^ **F]-FDG**	
Brain	3.09*	-	-
Head and neck	3.4±0.9	7.6±3.1	0.236
Lung	3.9±1.8	5.1±2.9	0.365
Mediastinum	7.6±1.3	7.7±1.8	0.904
Chest wall	9.1±1.8	7.8±2.3	0.537
Axillary	5.4±1.3	5.8±2.7	0.916
Liver and biliary system	21.3±2.3	9.4±2.1	0.116
Spleen	7.0*	11.8*	-
Pancreas	3.8*	-	-
Adrenal	4.0±0.1	3.2±1.0	0.504
Gastrointestinal tract	7.1±2.8	6.7±6.0	0.894
Genitourinary tract	6.4*	5.0*	-
Pelvis	1.5*	9.7±4.4	-
Musculoskeletal system	4.8±0.5	5.4±1.1	0.661

*: In body regions with a single metastasis, the net SUV_max_ is reported (not the mean±SD).

***Target to background ratio (TBR)***

 SUV_max_ was standardized according to the liver uptake, and the target to background ratio (TBR) was calculated. The average liver background SUV_max_ values for [^68^Ga]-FAPI-46 and [^18^F]-FDG PET/CT scans were 1.5±0.1 and 2.9±0.2, respectively (p=0.001). Also, the average blood pool SUV_max_ were 1.7±0.1 for [^68^Ga]-Ga-FAPI-46 PET/CT and 2.0±0.1 for [^18^F]-FDG PET/CT (p=0.016).

 A comparison of TBR between the two scans in different body regions revealed that the TBR was significantly higher in the lung, chest wall, mediastinum, and musculoskeletal areas for FAPI-46 PET/CT compared to [^18^F]-FDG PET/CT (Table 4).

**Table 4:** Target-to-background ratio (mean±SD) of detected lesions in different body regions in [^68^Ga]-Ga-FAPI-46 versus [^18^F]-FDG PET/CT scans

**Body region**	**Target-to-background ratio (mean±SD)**		**p-value**
	**[** ^68^ **Ga]-Ga-FAPI-46**	**[** ^18^ **F]-FDG**	
Brain	1.8*	-	-
Head and neck	1.8±0.5	2.4±1.0	0.640
Lung	3.0±1.7	2.1±1.3	0.033
Mediastinum	5.2±0.8	1.4±0.5	0.043
Chest wall	7.6±1.3	2.8±0.7	0.048
Axillary	4.7±1.3	2.1±1.0	0.230
Liver and biliary system	4.1±1.6	3.2±0.9	0.600
Spleen	3.7*	4.5*	-
Pancreas	3.2*	-	-
Adrenal	2.2±0.2	1.1±0.5	0.286
Gastrointestinal tract	4.7±1.5	2.0±0.8	0.150
Genitourinary tract	3.8*	1.5*	-
Pelvis	0.8*	3.7±2	-
Musculoskeletal system	3.3±0.3	1.9±0.3	0.03

*: In body regions with a single metastasis, the net target to background ratio is reported (not the mean±SD).

 No correlation was observed between the SUV_max_ or blood pool standardized SUV_max_ of either [^18^F]-FDG or [^68^Ga]-Ga-FAPI-46 results and the Ki-67 index. Among the receptors studied, only PR positivity correlated with standardized SUV_max_ of the lesions in both [^68^Ga]-Ga-FAPI-46 and [^18^F]-FDG imaging (p=0.005, rs=0.873). 

***Staging of patients***

 Patient staging, based on the TNM classification system, was consistent across both [^68^Ga]-Ga-FAPI-46 and [^18^F]-FDG PET/CT scans. In total, 9 patients (75%) were in stage IV, 2 patients (16.6%) were in stage 0, and 1 patient (8.3%) was in stage IIA of breast cancer.

 In more detail, the T component consisted of 10 cases of T0 (83.3%), one case of T4b (8.3%), and one case of T4c (8.3%). The N component was N0 in 7 cases (58.3%), N1 in 1 case (8.3%), N2 in 2 cases (16.6%), and N3 in 2 cases (16.6%). The M component consisted of 3 cases with M0 (25.0%) and 9 cases with M1 (75.0%).

## Discussion

 Breast cancer is the most prevalent cancer diagnosed and the foremost cause of cancer-related deaths among women (15). Common modalities used in the diagnosis and staging of breast cancer include mammography, breast ultrasound, MRI, and PET/CT (16). [^18^F]-FDG PET/CT plays a crucial role in the management of breast cancer patients, including diagnosis, initial staging, restaging, treatment response evaluation, and prognostication (3, 17). On the other hand, [^18^F]-FDG PET/CT has low sensitivity in detecting breast tumors smaller than one centimeter, micro-metastases, ductal carcinoma in situ, or lobular carcinoma due to either spatial resolution of the device or indolent nature of the lesions (18, 19). 

 Additionally, benign pathologies with increased [^18^F]-FDG uptake, including infections, fibroadenoma, ductal adenoma, granulomatous mastitis, and fibrocystic changes in the breast as well as post-operative inflammatory process, can reduce the specificity of the modality (6, 20).

 In the present study, we compared the efficiency of [^68^Ga]-Ga-FAPI-46 against [^18^F]-FDG PET/CT scans in breast cancer patients. We noted that the background uptake for the liver and spleen was markedly lower with [^68^Ga]-Ga-FAPI-46 compared to [^18^F]-FDG. While there was no significant discrepancy in the number of lesions detected and the SUV_max_ values of the lesions between the two modalities, the SUV_max_ ratio of the total lesions to the liver stood significantly elevated with [^68^Ga]-Ga-FAPI-46. This enhanced ratio was also evident in [^68^Ga]-Ga-FAPI-46 for lesions in the breast, chest wall, skeletal structures, and mediastinum. Importantly, [^68^Ga]-Ga-FAPI-46 imaging does not necessitate dietary regimens, preparations, or blood sugar monitoring. It can even be performed 30 minutes post-injection, presenting a more convenient alternative for both patients and imaging centers.

 In our study, there was no significant difference in the number of lesions detected between the two modalities. This contrasts with the findings of Novruzov et al., who compared [^68^Ga]-Ga-FAPI-46 and [^18^F]-FDG in breast cancer. In their research, FAPI identified more lesions in 11 out of 75 patients than FDG. This included a second lesion in the same breast, metastatic lymph nodes, and even a metastatic bone lesion that [^18^F]-FDG failed to detect (21). 

 While the total number of lesions did not display a statistically significant difference between the modalities, we identified lesions in 4 patients that either went unnoticed or appeared ambiguous on [^18^F]-FDG. These lesions were, however, clearly delineated on [^68^Ga]-Ga-FAPI-46 PET/CT scan. Specifically, one patient displayed 3 brain and 3 spinal lesions; another patient had 6 bone lesions, which encompassed 5 spinal lesions and 1 rib; a third patient showed an internal mammary lymph node; and the fourth patient had 8 bone lesions along with mediastinal lymph nodes. 

 The disparities observed between our findings and those of Novruzov can be attributed to the notably larger sample size in their study and the varied patient characteristics, since chemo-therapy might lead to a down-regulation of FAP receptor. In our cohort, 75.0% of patients had undergone surgery, and all had received chemotherapy. In contrast, none of the participants in Novruzov study had undergone any treatment. Altogether, a recent systematic review by Evangelista et al., involving 172 patients in 13 studies, revealed that FAPI PET imaging is superior to FDG PET imaging in breast cancer patients due to a lot of reasons including the ability to detect more lesions and the opportunity to detect small lesions after chemotherapy (22). 

 In our study, SUV_max_ of lesions did not show a significant statistical difference between the two modalities, but the overall SUV_max_ ratio of lesions to the liver in [^68^Ga]-Ga-FAPI-46 was significantly higher than [^18^F]-FDG. This ratio was also significantly higher in [^68^Ga]-Ga-FAPI-46 for lung, chest wall, bone, and mediastinal lesions. In contrast to our study, Novruzov et al. reported that the uptake of [^68^Ga]-Ga-FAPI tracer in primary lesions was significantly higher than [^18^F]-FDG, and this higher uptake was also observed in lymph nodes. Their study showed no correlation between [^68^Ga]-Ga-FAPI uptake and pathological grade, Ki-67 index, or patient age. Despite our results differing from Novruzov et al. in terms of SUV_max_ values between [^68^Ga]-Ga-FAPI and [^18^F]-FDG, the tumor-to-background ratio, in general, was higher in [^68^Ga]-Ga-FAPI, offering improved contrast and easier lesion detection.

 Enhanced tumor-to-background ratios with [^68^Ga]-Ga-FAPI were also reported by other researchers. For instance, Wegen et al., investigating head and neck cancers, found consistently higher [^68^Ga]-Ga-FAPI uptakes in tumoral lesions compared to the background than [^18^F]-FDG PET/CT results (23). Another study by the same team on cervical cancers confirmed these findings, presenting significantly greater tumor-to-background contrast with FAPI for detecting tumor lesions (24).

 In the study conducted by Komek et al., which examined [^68^Ga]-Ga-FAPI-04 in breast cancer, it was reported that the radiopharmaceutical had superior detection ability for primary breast lesions as well as liver, bone, lymph node, and brain metastases (11). These findings were consistent with our study, particularly in relation to lesions in the lung, chest wall, bones, and mediastinum. Additionally, in this study, SUV_max_ of primary breast tumors, lymph nodes, lung metastases, and bone metastases were higher in [^68^Ga]-Ga-FAPI compared to [^18^F]-FDG. However, according to the mentioned report, there was no difference in [^68^Ga]-Ga-FAPI and [^18^F]-FDG uptake in liver metastases, which is similar to our study. 

 [^68^Ga]-Ga-FAPI also resulted in a significantly higher tumor-to-background ratio in breast lesions, liver, bone, brain, and lung metastases, but this higher ratio was observed only in the lung, chest wall, mediastinum, and skeletal muscles in our study.

 Although our data did not reveal [^68^Ga]-Ga-FAPI-46 SUV_max_ as surpassing that of [^18^F]-FDG, the enhanced tumor-to-background ratio in specific anatomical areas points to an improved lesion visualization, even in lesions with low uptake. Given these results and the robust biological rationale, [^68^Ga]-Ga-FAPI-46 remains a promising candidate for future research endeavors.

 Similarly, a 2022 systematic review by Li et al. highlighted the superiority of tumor-to-background ratio and better detection of bone metastases of [^68^Ga]-Ga-FAPI over [^18^F]-FDG (25). This finding, along with the strong biological background, still makes [^68^Ga]-Ga-FAPI one of the potential research options for future studies.

 Our observations concerning [^68^Ga]-Ga-FAPI-46 uptake in the lesions with no [^18^F]-FDG uptake could have significant implications for treatment response assessment. Contrasting [^18^F]-FDG, which identifies pathologies through elevated glycolysis, [^68^Ga]-Ga-FAPI binds to distinct cancer cell surface targets and binds onto tumor-associated fibroblasts until connecting to neighboring normal tissues. 

 Given that FAP is a glycoprotein transmembrane receptor found on cancer-associated fibroblasts (CAFs), differentiating between tumor and non-tumor lesions may be more effective using this method.

 It seems that [^18^F]-FDG may have a higher false-positive rate since it is involved in metabolism. For example, after surgery, granulation tissue may absorb [^18^F]-FDG, but not [^68^Ga]-Ga-FAPI. However, false-positive findings have also been reported in [^68^Ga]-Ga-FAPI PET studies, including Schmorl nodes in bones, thyroiditis, hemangiomas, and pneumonia, 

as well as fibrous tumors or chronic cystitis (generally chronic inflammation). It's worth noting that most such lesions aren't typically confused with primary or metastatic tumors. Moreover, lactating breast might have FAPI uptake, which is diffuse and not confusing with tumoral uptake (26). Furthermore, [^68^Ga]-Ga-FAPI PET/CT may be useful in patients undergoing radiation therapy, as [^18^F]-FDG PET/CT may not perform well in distinguishing between acute inflammation after radiation and residual malignant disease. Conversely, [^68^Ga]-Ga-FAPI PET/CT shows high uptake in chronic inflammation processes, but generally does not show significant uptake in acute inflammatory processes, facilitating more straightforward differentiation of tumor residues (27-32). 

 Nonetheless, these observations require validation through larger, more robust studies. In the present study, both the PET modalities presented the similar disease staging and TNM classification. [^68^Ga]-Ga-FAPI PET/CT findings thus can be useful in presenting the accurate disease staging. As in the present study, [^68^Ga]-Ga-FAPI PET/CT identified more lesions and these findings may be of potential utility in breast cancer which however, needs validation in a larger number of patients. For example, in a study on pancreatic adenocarcinomas, among 19 patients, [^68^Ga]-Ga-FAPI PET/CT changed the stage in 10 patients, with 8 cases being upstaged and 2 cases being down staged (33). 

 Another research on gastrointestinal tumors highlighted that [^68^Ga]-Ga-FAPI restaged 46.9% of cases, subsequently altering treatment plans for 25% of them (34).

 The high uptake and tumor-to-background ratio of [^68^Ga]-Ga-FAPI might suggest that a beta-emitter FAP-based compound could potentially be an effective treatment strategy for tumors with high FAP expression. This could be a new step in breast cancer treatment in the future.

 The main limitation of our study was the small sample size, which produced weak statistical power compared to similar studies with larger sample sizes; however, the sample size range in the previous, systematic review (22) spans from 2 to 48 (median=12), akin to our study's sample size. In future studies, it is recommended to conduct dosimetry to investigate radiation exposure to non-target organs and tissues, increase the sample size to investigate the diagnostic accuracy of [^68^Ga]-Ga-FAPI imaging, and follow up the patients especially in cases where the lesion does not show [^18^F]-FDG uptake.

## Conclusion

 Both, [^68^Ga]-Ga-FAPI-46 and [^18^F]-FDG PET/CT demonstrated identical detection efficiency, SUV_max_ values and disease staging. With the observed significantly lower activity in the blood pool and the liver, [^68^Ga]-Ga-FAPi PET/CT provided better image contrast and lesion detectability. This was more notable in the mediastinal and skeletal lesions. These findings may thus pave the way for the development of effective FAP based radioligand therapies using beta emitters in advanced-stage breast cancers.
